# Supplementary material for: Experiences of living with mental health problems during the COVID-19 pandemic in the UK: a coproduced, participatory qualitative interview study
Source: Soc Psychiatry Psychiatr Epidemiol. 2021 Mar 4;56(8):1447–57. doi: 10.1007/s00127-021-02051-7 (PMC7931976; doi:10.1007/s00127-021-02051-7)
Supplement: Supplementary file 1 — Supplementary file1 (DOCX 42 KB) [file 127_2021_2051_MOESM1_ESM.docx]

**Experiences of living with mental health problems during the COVID-19 pandemic in the UK: a coproduced, participatory qualitative interview study**

**Interview topic guide**

**Journal: Social Psychiatry and Psychiatric Epidemiology**

**Steven Gillard, PhD^1^**

**Ceri Dare, MPH^2^**

**Jackie Hardy, DipBM^2^**

**Patrick Nyikavaranda, MSc^2^**

**Rachel Rowan Olive, GCert^2^**

**Prisha Shah, BA^2^**

**Mary Birken, PhD^2^**

**Una Foye, PhD^3^**

**Josephine Ocloo, PhD^3^**

**Ellie Pearce, PhD^2^**

**Theodora Stefanidou, MSc^2^**

**Alexandra Pitman, PhD^2^**

**Alan Simpson, PhD^3^**

**Sonia Johnson, MD^2^**

**Brynmor Lloyd-Evans, PhD^2^**

**On behalf of the NIHR Mental Health Policy Research Unit Covid coproduction research group**

1. Centre for Mental Health Research, City, University of London, 1 Myddelton Street, London EC1R 1UW

2. Division of Psychiatry, University College London, Maple House, 149 Tottenham Court Road, London W1T 7NF

3. Institute of Psychiatry, Psychology and Neuroscience, King’s College London, 18 DeCrespigny Park, London SE5 8AF

Corresponding author: Brynmor Lloyd-Evans

Address: Division of Psychiatry UCL, Maple House, 149 Tottenham Court Road, London W1T 7NF

Email: [b.lloyd-evans@ucl.ac.uk](mailto:b.lloyd-evans@ucl.ac.uk)

Tel: 00 44 (0)20 7679 9428

ORCID ID: 0000-0001-9866-788X

**Title of Study:** Exploring the lived experiences of loneliness and isolation with people with mental health problems during the COVID-19 pandemic in the UK.

**Interview Topic Guide V2, 16/04/20**

**(With clarified demographic questions and interviewer prompts)**

**Pre-interview checklist**

- Confirm with the researcher who set up the interview any input you want from them (e.g. they could help with taking consent and answering questions about the study)
- Confirm with the researcher recording how you will contact them if needed during interview
- Confirm with the researcher organising the interview how you will check in with them after the interview

Have a copy to hand of:

- Participant Information Sheet
- Consent Form
- Topic Guide
- List of resources that may be helpful to refer to if the persons is distressed, including details for: Samaritans, CALM, MIND

**Starting the interview: interviewer prompts:**

- **Introduce self and person recording and explain role.**
- **Check the participant is happy with the interview technology. If they are participating online, remind them they have the choice to have their video on or off.**
- **Ask the participant if they have read the Participant Information Sheet, and if they have any questions.**
- **Capacity to consent questions: ask about aspects of Participant Information Sheet (refer to slides and decide which questions you would like to ask).**
- **(If this has not previously been discussed and started), confirm with the participant that the researcher will start recording the interview now – i.e. in all cases before taking formal consent**
- **Complete the Consent Form by reading out each statement and asking person to verbally agree after each statement.**

**Introduction**

Thank you so much for agreeing to be interviewed.

As you know the purpose of the interview is to find out about your experiences during the virus outbreak, and more generally, your experiences of feeling lonely or isolated and how they may relate to experiences of mental health problems. This will help us understand more about mental health, isolation and loneliness and how it relates to the virus outbreak. It will also help us to do further research looking at how to measure these things, or to develop ways of supporting people’s mental health and reducing loneliness and isolation particularly during virus outbreaks. We will also learn more about the other challenges you have faced during the virus outbreak and what can be helpful for people experiencing mental health problems during this new situation.

There are no right or wrong answers, as everyone’s experience will be different. What you say will be kept completely confidential and we’ll anonymise the information you give us, so we won’t use your name or say anything that could identify you, especially in anything we write. Please be as open as you feel comfortable, and if you want to skip a question or take a break at any point, please let me know.

Firstly, I’d like to ask you for some information about yourself, so we can understand the context of your answers in relation to the virus.

- Are you currently using mental health services **Y/N**
- If no, have you ever? **Y/N**; when was your most recent contact?
- What is the current/most recent types of service you have used?
  - Who do you currently live with: **partner, children, other family, flatmates, alone**. (select all that apply)
  - Has this changed due to Covid **Y/N** (who did you live with before)
  - Are you in paid work? **i) Yes, currently working; (at home; or in a workplace?) ii) Yes but furloughed (currently not working but having your wages paid through the government scheme and/or your employer); iii) No**
  - If yes: **i) Full time; or ii) part-time**
  - Are you volunteering? **Yes/No** (If yes, please describe)
  - Are you in education? **Yes/No** (If yes, please describe)
- What region of the UK do you live in? **[1. North East, 2. North West, 3. Yorkshire and Humber, 4. West Midlands, 5. East Midlands, 6. East of England, 7. London, 8. South East 9. South West]**
- What kind of environment do you live in: **City over 100,000, smaller city or town, village, countryside/rural**
- The public are being asked to stay at home as much as possible (that is, only leaving the house for food shopping, exercise or work if you can’t work at home). How long have you been limiting your social contact with others in this way? **Haven’t been, or duration in weeks**
- Have you had any COVID 19 virus symptoms, or have you been diagnosed with the virus? **i) Yes, definitely; ii) Probably but unsure; iii) Not as far as I know**
- Are you shielding at home on advice from government, or currently self-isolating because of your symptoms, or someone else’s symptoms? **i) Shielding to avoid getting the virus; ii) self-isolating because of my symptoms; iii) self-isolating because of others’ symptoms**

**Interview questions**

**1. Can you tell me about the main impact of the virus outbreak on you?**

Prompts: day to day impact on routine, who you see or have any contact with, practical issues, access to services and support, going outside and whether any difficulties arise when you do. Impact on relationships with family/friends.

**2. (For those using mental health services) How has the care you receive been affected by the virus outbreak?**

Prompts: what service did they receive before, i.e. how often did they see someone from service, was it face to face, was it in outpatients, or at home? What has changed, or stopped? Has it changed mid treatment/therapy? How have they found adapting to any changes from face to face contact? Was there any choice or consultation about how the changes to your mental health care were arranged?

**3. Do you feel isolated or lonely currently, during this “stay at home”, lockdown time?**

**Prompts:** Have you felt more or less lonely during the virus outbreak?

How does loneliness impact on you day to day during the virus outbreak/ and before the virus outbreak?

**4. Can you tell me about how your mental health has been since the virus outbreak has developed? What about since the lockdown “stay at home” advice has been put in place?**

Prompt: tell me more about the changes. Any things which have got more difficult, new difficulties? Any positive effects of the virus outbreak and staying at home on your mental health?

**5. How are you coping with your mental health in the current situation?**

Prompts: has anything helped? Has anything you tried, or any support you were offered, not worked?

Any barriers to accessing new options available, ie when people live with lots of people, is privacy for phone calls to services difficult, finance to make calls.

What support or guidance on mental health and the virus have you read or heard about? (e.g. via social media or websites, from leaflets, or on TV, from any mental health charities like MIND, or NHS?)

What guidance have you followed?

Have you found it clear, has it been helpful? [Explore: managing social distancing and knowing when you can go out; hygiene measures e.g. handwashing, wearing masks]

**Interviewer prompt: That’s the end of the questions relating to the virus outbreak. Thank you for sharing your experiences. Now I would like to ask you about experiences of loneliness in your life more generally, not just in relation to the current virus outbreak. But I wanted to pause here and ask, how have you found the interview so far?**

**Are you ok to continue with the interview?**

**Would you like to take a break?**

**Would you like to do the next part on another day?**

**6. Experience of loneliness**

**Can you tell me what the word lonely means to you? How would you define it?**

Interviewer Prompts:

- How does it make you feel?

Physically, psychologically, emotionally e.g. social anxiety.

**7. Has there been a time or times in your life when you have been lonely?**

**Would you say you feel lonely in your life in general at the moment?**

**What is/was that like?**

Interviewer Prompts:

- Are there things that you think have triggered or underlie your feeling of loneliness? E.g. age, culture, personality style, , difficulty fitting in, stopping work, moving location, loss of a partner or family.
- Are there situations or times when you feel more lonely than others? Prompt: e.g. seasonal, not working.

**Social contact and loneliness**

**8. Do you feel lonely when you are in the company of others?**

**Are there any kinds of social contact that make you feel more or less lonely**?

Interviewer Prompts:

- E.g. groups, family, special friend, in person rather than online or using technology.
- When you meet people in person, do you feel that you can talk to them easily?

**9. Do you think that spending time on your own can be helpful/therapeutic?**

**10. Does spending too much time on your own negatively affects your wellbeing?**

- [If yes] - How much is too much? Where is the line?

**Loneliness and mental health**

**11. Do you think feeling lonely is connected to your mental health?**

- [if yes]: In what ways?

Interviewer Prompts:

- Does feeling lonely make your mental health worse? In what ways?
- Do you think your mental health problems or your treatment contribute to your loneliness? [Prompts: e.g. side effects of medication, the age you developed mental health problems.]
- Thinking back to when your mental health problems first started, do you think that feeling lonely came before or after?
- [If loneliness came first]: Has your experience of loneliness changed since having mental health problems?

**12. Do you feel you belong in the community around you?**

- Prompts: explore different communities: neighbourhood, family and friendship groups, communities of interest [If not]: Can you tell me more about this feeling of not belonging? In what ways, if any, does that relate to your feeling of loneliness?

**Final questions:**

**13. Are there ways in which you have tried to reduce your loneliness? What has and has not worked?**

Interviewer Prompts:

- Do you have ways of coping?

Have you had any support? What kind of support? Has this helped?

**14. What would not being lonely look like for you?**

Interviewer Prompts:

- How do you imagine your life would be different?

**15. What tips or advice would you give someone who was struggling with loneliness?**

Finally, I’m going to ask you for some information about yourself. We are collecting this so that we can make sure we interview a range of people, and to help us provide some context to what you say. If there are any questions you would prefer not to answer, please just ask the researcher to move on to the next question: you don’t have to provide any information you don’t feel comfortable sharing.

- How would you prefer to describe your gender: (read all out and person can then answer)

**1. Male**

**2. Female**

**3. Another way (If in another way, please tell us what term you prefer)**

**4. Prefer not to say**

- Do you consider yourself to be a trans person? **(Yes, No, Prefer not to say)**
- Age (in years)
- How would you describe your ethnicity?

**White**

- English / Welsh / Scottish / Northern Irish / British
- Irish
- Gypsy or Irish Traveller
- Any other White background

**Mixed / Multiple ethnic groups**

- White and Black Caribbean
- White and Black African
- White and Asian
- Any other Mixed / Multiple ethnic background

**Asian / Asian British**

- Indian
- Pakistani
- Bangladeshi
- Chinese
- Any other Asian background

**Black / African / Caribbean / Black British**

- African
- Caribbean
- Any other Black / African / Caribbean background

**Other ethnic group**

- Arab
- Any other ethnic group
- First language
- Which of the following best describes your sexual orientation: **i) Heterosexual/straight; ii) Bi/bisexual; iii) Gay/lesbian; iv) Prefer not to say**
- Do you consider that you have a disability **Y/N**

Medication prescribed for mental health condition(s) **Y/N.**

**SEE NEXT PAGE FOR END OF INTERVIEW PROMPTS**

**At the end of the interview - Interviewer prompts:**

- Ask the person how they found the interview and if they found any questions upsetting. Ask if they feel they would have anyone to talk to about this if they felt upset a few days later.
- Ask if they would like follow-up phone call or email as few days later to check how they are and sign post to resources. [This may be from a study researcher rather than you the interviewer.]
- Ask if they would like list of resources and mention any ones that they may specifically find helpful.
- Remind them a researcher will be in touch about sending vouchers to say thank you for their time

**After the interview:**

- If you have any concerns for the participant’s or others’ safety, please talk to researcher about this during check in.
- If the participant wanted a follow-up contact, please clarify with the researcher organizing the interview if they should do this.
