# Supplementary material for: Experiences of living with mental health problems during the COVID-19 pandemic in the UK: a coproduced, participatory qualitative interview study
Source: Soc Psychiatry Psychiatr Epidemiol. 2021 Mar 4;56(8):1447–57. doi: 10.1007/s00127-021-02051-7 (PMC7931976; doi:10.1007/s00127-021-02051-7)
Supplement: Supplementary file 3 — Supplementary file3 (DOCX 31 KB) [file 127_2021_2051_MOESM3_ESM.docx]

**Experiences of living with mental health problems during the COVID-19 pandemic in the UK: a coproduced, participatory qualitative interview study**

**Interview topic guide**

**Journal: Social Psychiatry and Psychiatric Epidemiology**

**Steven Gillard, PhD^1^**

**Ceri Dare, MPH^2^**

**Jackie Hardy, DipBM^2^**

**Patrick Nyikavaranda, MSc^2^**

**Rachel Rowan Olive, GCert^2^**

**Prisha Shah, BA^2^**

**Mary Birken, PhD^2^**

**Una Foye, PhD^3^**

**Josephine Ocloo, PhD^3^**

**Ellie Pearce, PhD^2^**

**Theodora Stefanidou, MSc^2^**

**Alexandra Pitman, PhD^2^**

**Alan Simpson, PhD^3^**

**Sonia Johnson, MD^2^**

**Brynmor Lloyd-Evans, PhD^2^**

**On behalf of the NIHR Mental Health Policy Research Unit Covid coproduction research group**

1. Centre for Mental Health Research, City, University of London, 1 Myddelton Street, London EC1R 1UW

2. Division of Psychiatry, University College London, Maple House, 149 Tottenham Court Road, London W1T 7NF

3. Institute of Psychiatry, Psychology and Neuroscience, King’s College London, 18 DeCrespigny Park, London SE5 8AF

Corresponding author: Brynmor Lloyd-Evans

Address: Division of Psychiatry UCL, Maple House, 149 Tottenham Court Road, London W1T 7NF

Email: [b.lloyd-evans@ucl.ac.uk](mailto:b.lloyd-evans@ucl.ac.uk)

Tel: 00 44 (0)20 7679 9428

ORCID ID: 0000-0001-9866-788X

**DS3**

**Organisations contacted during participant recruitment to publicise the study**

In addition to publicising the study nationally through social media, researchers contacted the following organisations to help recruit participants. Information about the study was disseminated via posters, or via email or other means to organisations’ members/service users.

| **Organisation** | **Description** |
| --- | --- |
| **Archway Foundation** | An Oxford-based voluntary sector organization offering social spaces and 1:1 support for people affected by loneliness |
| **Black Thrive** | A South-London based organization aiming to improve the prevention and treatment of mental illness in the Black community and the social causes behind it, and connect people and organisations to achieve change in the community |
| **Carribbean and African Health Network** | A Black-led organisation set up to address the wider social determinants to support community resilience and reduce health inequalities for people of Caribbean & African in Greater Manchester and beyond |
| **Dragon Cafe** | A survivor-led organization in South London providing a creative and supportive space to promote mental health recovery and address social isolation |
| **Marmalade Trust** | A Bristol-based community organization which aims to find and support isolated people to reconnect with society and enjoy better lives. |
| **Mind in Harrow** | A North London branch of the national mental health charity MIND, providing a range of support and services for people with mental health conditions. |
| **National Survivor User Network** | A national user-led network which aims to create and strengthen links between individuals and groups, support and promote user-led groups and initiatives, and influence and inform policy and decision makers |
| **One Housing** | A London-based Housing Association providing housing and a large homeless shelter |
| **Rosetta Arts** | An East London-based arts organization which aims to engage local communities and venues, discovering and supporting artists at different stages of their artistic development |
| **Southwark Association for Mental Health** | A user-run mental health charity based in South London which provides mental health support and campaigning, including supporting the Kindred Minds project for people from Black and ethnic minority communities. |
